# Effect of simulated brushing on surface roughness and wear of bis-acryl-based materials submitted to different polishing protocols

**DOI:** 10.4317/jced.58920

**Published:** 2022-02-01

**Authors:** Rafael-Francisco-Lia Mondelli, Lorena-de Mello-Alcântara Garrido, Ana-Flávia Soares, Allison-Danitza Rodriguez-Medina, José Mondelli, Fernanda-Sandes de Lucena, Adilson-Yoshio Furuse

**Affiliations:** 1 DDS, MSc, PhD. Department of Operative Dentistry, Endodontics and Dental Materials, Bauru Dental School, University of São Paulo, Bauru, Brazil; 2 DDS, MSc. Department of Operative Dentistry, Endodontics and Dental Materials, Bauru Dental School, University of São Paulo, Bauru, Brazil; 3 DDS, MSc, PhD. Department of Health, State University of Southwestern Bahia, Jequié, Brazil; 4 DDS. Department of Operative Dentistry, Endodontics and Dental Materials, Bauru Dental School, University of São Paulo, Bauru, Brazil

## Abstract

**Background:**

Provisional materials must have enough strength to withstand masticatory loads without suffering deformation or fracture, and their surfaces must have good finishing and polishing characteristics to reduce biofilm accumulation. Thus, the purpose of this study was to investigate the best polishing protocol for different bis-acryl composite resins in comparison with a conventional resin composite and a self-curing acrylic resin aiming to obtain a smooth restoration surface and resistance to wear.

**Material and Methods:**

One hundred and four samples (15 mm length x 5 mm width x 4 mm depth) were prepared and divided into four groups according to the material tested: Protemp 4 and Structur 3 bis-acryl composite resins, Dencor self-curing acrylic resin, Filtek Z350XT conventional composite resin. The polishing procedures were performed with Sof-Lex Pop-On discs or Sof-Lex spirals and abrasion procedures were performed on a brushing machine. The surface roughness was analyzed at three periods (initial, post-polishing and post-brushing) and the wear was evaluated after simulated brushing. The results were submitted to ANOVA followed by the Tukey (α = 0.05).

**Results:**

Filtek Z350XT groups showed the lowest values of initial surface roughness followed by Structur 3, Protemp 4 and Dencor groups. After polishing and simulated brushing, Filtek Z350XT groups also presented the lowest roughness values, followed by bis-acryl groups (Structur 3 and Protemp 4) and Dencor groups demonstrated the highest surface roughness. Sof-Lex Pop-On discs system exhibited lower roughness values for all groups.

**Conclusions:**

Sof-Lex Pop-On discs system promoted the best polishing for all groups. Overall, Filtek Z350XT groups presented lower results for both roughness and wear for all periods evaluated, followed by Protemp 4 and Structur 3, meanwhile Dencor groups presented the highest roughness and wear values for all periods.

** Key words:**Acrylic resin, bis-acryl, brushing, composite resin, polishing, roughness, wear.

## Introduction

Provisional restorations play an essential role in fixed prosthodontics. They are manufactured prior to the installation of ceramic laminate veneers and crowns, providing the maintenance of periodontal and pulpal health, reestablishment of aesthetics and occlusal function and also guide the clinician regarding color, shape, contour and proximal contacts of the definitive restoration (1,2). Ideally, the selected temporization material should present good mechanical and physical properties, as well as easy handling and biocompatibility to oral tissues (3).

The most frequent materials used for these restorations are acrylic resins, such as polymethyl methacrylate (PMMA), polyethyl methacrylate (PEMA), and composite resins (4,5). Notwithstanding the cost-effectiveness of PMMA and PEMA materials, drawbacks like low mechanical properties, poor color stability, high exothermic polymerization and large shrinkage, which can lead to injuries to pulp tissue and restoration failures (6).

In the past decade, bis-acryl-based resins, composed of bisphenol A-glycidyl methacrylate (Bis-GMA), urethane dimethacrylate (UDMA) and inorganic fillers were introduced aiming to facilitate the manufacturing of provisional restorations, improving color stability, strength and esthetics, overcoming the issues of the methacrylate-based materials (7-9). The bi-functional substrates of this interim material are responsible for the formation of a cross-linked monomer chain, increasing flexural strength and toughness and the filler particles enhance abrasion resistance (10). Bis-acryl resins are presented as cartridges or syringes that can be mixed through an auto mixing tip, facilitating the material handling and allowing the use for carrying out mock-up restorations and demonstrate the predictability of the definitive restorative treatment (11).

The provisional phase of restorative treatment can be considered successful when it remains in the mouth the appropriate time for the completion of the final prosthesis or veneers without causing damage to oral tissues (12). However, excessive surface roughness facilitates biofilm accumulation leading to inflammation (8), bleeding and gingival recession (11). To improve longevity and quality of interim materials, remove excess of restoration, reduce surface imperfections and produce a smooth and glossy surface, less likely to plaque retention, finishing and polishing procedures are recommended (13). Despite different polishing techniques and materials available, such as rubberized discs or spirals, abrasive silicone tips (14), diamond and aluminum oxide pastes or pumice (15), few manufacturers present instructions on the most advantageous polishing method for their products.

Besides the several published reports respecting mechanical properties of provisional materials (5,16-18), less is known about the proper polishing procedures and resistance to wear after brushing, especially for bis-acryl materials, since some manufacturers (i.e. Structur 3, VOCO) stated that only alcohol wiping on the surface is sufficient to provide a smooth surface. Accordingly, the purpose of this study was to investigate the best polishing protocol for different commercially available bis-acryl resins in comparison to acrylic and composite resins to determine the surface roughness modification and abrasive wear after simulated brushing. The null hypothesis tested was that the material type and polishing technique do not affect surface roughness and resistance to wear after simulates brushing of the materials under study.

## Material and Methods

This *in vitro* study involved 3 factors: provisional material at 4 levels, 2 bis-acryl resins (Structur 3 and Protemp 4), 1 acrylic resin (Dencor) and 1 composite resin (Filtek Z350XT), polishing materials at 2 levels (Sof-Lex Pop-On discs and Sof-Lex spirals) and time at 3 levels (initial, post polishing and post simulated brushing) with responsible variables of surface arithmetic roughness (Ra, µm) and wear (µm). The materials information is displayed in Table 1.

**Table 1:**
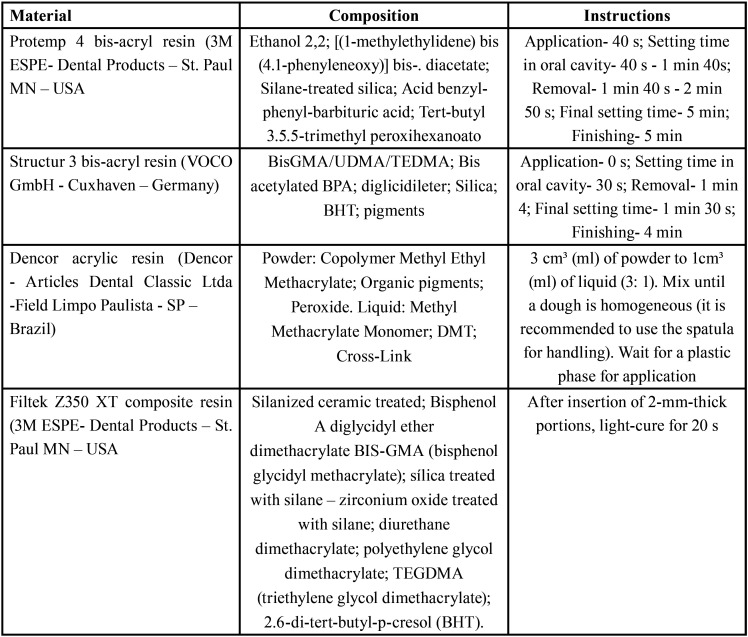
Materials used in the present study.

One hundred and four specimens (n=13) were obtained (Table 2). For each completed step (i.e. moment during sample preparation), one sample of each group was randomly selected for Scanning Electron Microscopy analysis (SEM, JSM – T220A - JOEL Ltda., Tokyo, Japan) resulting in 10 samples for each group (n=10). Bar-shaped samples of 15-mm-length, 5-mm-width, and 4-mm-depth were prepared using a specific stainless steel matrix (19).

**Table 2:**
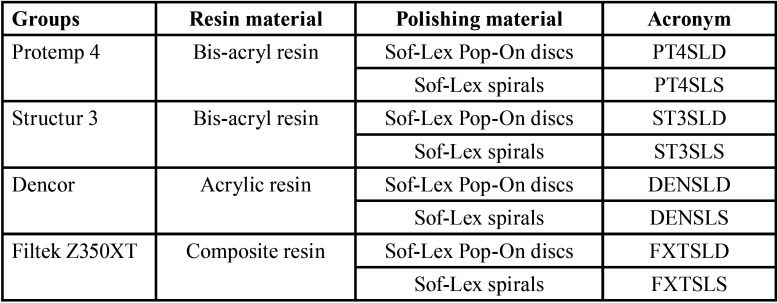
Groups according to the materials and polishing techniques.

Single increments of the bis-acryl resins were inserted into the mold and pressed (500 g) to obtain a flat surface with the support of an addition silicone matrix (Adsil Putty Soft – VIGODENT, Rio de Janeiro, RJ, Brazil) following the manufacturer’s instructions. After curing, ethyl alcohol was wiped on the surface with gauze for removing the superficial inhibition layer.

The acrylic resin was handled according to the manufacturer’s instructions (3:1 powder to liquid by volume) and set in a single increment with a spatula in the mold. To avoid the occurrence of bubbles, a gypsum dental vibration device was used during the handling and insertion of the acrylic resin, followed by pressing with a metallic presser.

The insertion of the composite resin was performed through the incremental technique with a 2 mm thickness for a total of four increments. A polyester strip (TDV Dental LTDA – Pomerode, SC, Brazil) was positioned on top of the final increment of the composite resin, and light-cured using a LED device (DB685 model Curing Light - Dabi Atlante - Ribeirão Preto, SP, Brazil) for 20 s with the tip of the device positioned as close as possible to the composite resin (20). Samples were then fixed on an acrylic disk and had their surfaces standardized in a polishing machine (Arapol 2V, Arotec, Cotia, SP, Brazil), using sandpapers with different granulations: 320, 400, 600 and, 1200 (Extec Corp. – Enfield, USA), and a felt disc (Extec Corp. – Enfield, USA) moistened with 1 μm diamond suspension (Erios – Equipamentos Ltda., São Paulo, SP, Brazil) for 4 minutes at high-speed with the same weight previously used.

After preparation, samples were immersed in deionized water and submitted to ultrasonic vibration (T7 Thorton, Unique Electronic Products Ltda., São Paulo, SP) for 5 minutes, followed by surface drying with absorbent paper.

After preparation and polishing, the means of three readings of surface roughness were made with a rugosimeter (Hommel Tester T1000 basic – Hommelwerke GmbH – ref #240851 - Schwenningem, Germany) and the mean for arithmetic roughness was obtained (Ra, µm). This parameter indicates the arithmetical average value of all differences in absolute distances of the roughness profile (R) from the centerline within the measuring length. The following parameters were used.

Minimum T = 0.01µm

Maximum T = 0.8µm 

Lt = 10 mm Lc = 0.25mm (cut-off) 

Lm = 4.5mm

Where.

T = tolerance (extreme values to be considered in readings)

Lm = measuring limit (considered extension of reading) 

Lt = trail limit (actual extent covered by the measuring probe tip) 

Lc = cut-off (filtering to minimize the surface ripple interference)(20)

All the samples were immersed in distilled water solution for 24 hours at 37°C.

Each material was submitted to two types of polishing.

1.Sof-Lex Pop On discs: polishing was conducted by positioning the samples on a scale (Electronic Kitchen scale - SF - 400), with controlled weight varying between 150 and 300 g using a low-speed handpiece (Dabi Atlante - N270 at 10.000 rpm) for 20 s for each grain (one-way movements). Between each sandpaper change, the samples were cleaned in a T7 Thorton ultrasound device (Unique Ind. and Com. of Electronic Products Ltda., São Paulo, SP) with a frequency of 40 kHz for 5 minutes with distilled deionized water and dried with absorbent paper.

2.Sof-Lex spirals: all samples followed the same procedure described above.

For the abrasion test, half of the samples (control side) were protected with an insulation tape and the test side was submitted to 50.000 brushing cycles on a simulated brushing machine (MN São Carlos, SP, Brazil), utilizing Essential Clean dental brushes (Colgate Palmolive Ind. Ltda., São Bernardo do Campo, SP, Brazil), at 37 ± 1oC and 300 g load. Every 120 s, 0.4 ml of slurry was injected. The slurry solution was prepared using Colgate Triple Action toothpaste (Colgate Palmolive Ind. Ltda., São Bernardo do Campo, SP, Brazil) and distilled water at a ratio of 1:2 by weight (19-22).

Three readings were performed for each sample, only on the brushed side, following the same parameters utilized in the initial surface roughness.

The rugosimeter was also used as a profilometer to determine the surface wear.

The parameters used were.

T minimum = 8 μm

T maximum = 40 μm

Lt = 10 mm; Lc = 0.00 mm (cut-off)

Lm = 9 mm

The samples were positioned and the reading was made perpendicular to the direction of movement of the simulated brushing. The spherical probe tip runs through the control side (unbrushed) to the brushed side, determining the wear in micrometers (μm) (20). Three readings were performed for each sample.

Three samples from each group (before and after polishing, and after wear) were randomly selected to be submitted to SEM for qualitative analysis. The samples were kept at room temperature for 12 h, then were mounted on aluminum stubs, fixed with colorless nail polish (Risque, Niasi, Taboão da Serra, SP, Brazil) and metalized with gold-palladium on a metallizer (DentronVacuum, Desk IV Moostestonn, NJ, USA), prior to SEM observation (JSM-T220A - JOEL Ltda., Tokyo, Japan), with 200x magnification.

Roughness data were submitted to three-way Anova, considering resin-based material, polishing technique and wear as independent variables. For surface wear, data were submitted to two-way Anova, considering resin-based material and polishing technique as independent variables. Multiple comparisons were performed by Tukey´s Test. A level of significance of 5% was used for all tests.

## Results

Mean values and standard deviations of roughness (initial and after polishing and brushing) and wear (after brushing) are shown in Table 3.

**Table 3:**
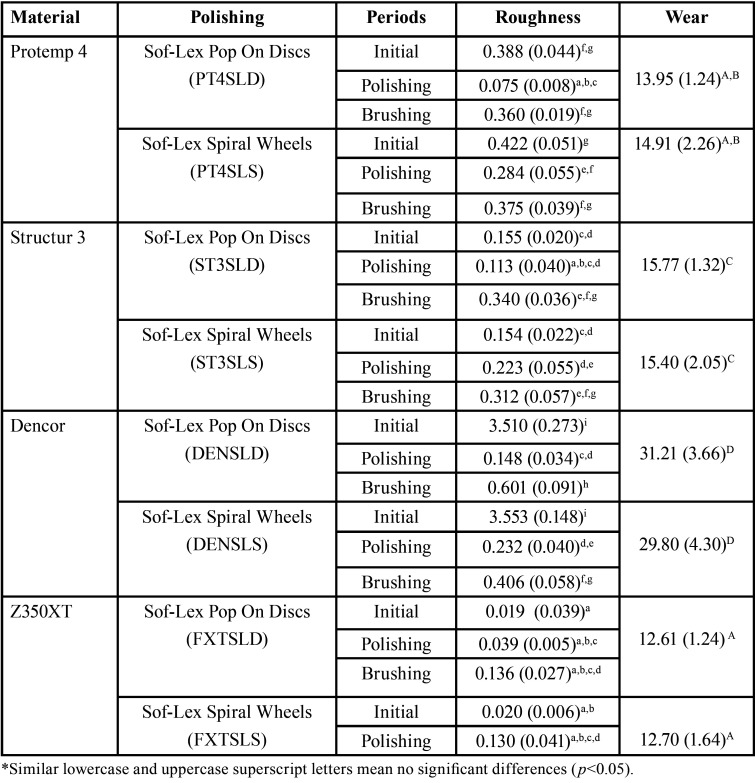
Mean and standard deviation of polishing, roughness and wear of the different tested materials.

Regarding surface roughness, for the PT4SLD group, there was a statistical difference between the initial (0.388 μm) and after polishing Ra (0.075 μm). After brushing, the Ra increased and presented a significant statistical difference when compared to the polishing (*p* < 0.05). The PROTSLS group showed a significant statistical difference between the initial Ra (0.422 μm) and Ra after polishing (0.284 μm). After brushing, the Ra values increased (0.375 μm), presenting a statistically significant difference when compared to the polishing, but did not present a statistically significant difference when compared to the initial Ra value (*p* > 0.001). The polishing with SLD resulted in lower roughness than the SLS (*p* < 0.05).

The ST3SLD group presented no statistically significant difference between the initial Ra (0.154 μm) and Ra after polishing (0.113 μm). After brushing, Ra values increased (0.340 μm) and there were differences when compared to the initial Ra (*p* > 0.05). For the ST4SLS group, there were no statistical differences between the initial Ra and after polishing, (*p* > 0.05). After brushing (0.312 μm), there was a statistically significant difference when compared to the initial Ra values (*p* < 0.05). When comparing the two bis-acryl resins, higher initial roughness was observed for PT4 (*p* < 0.05), independent of the polishing technique.

SLD polishing system showed lower Ra values for the two bis-acryl groups (ST3 and PT4), compared to SLS. However, there was a statistical difference only between the polishing procedures for PT4 group (*p* < 0.05).

After brushing, the PT4 groups, for the two types of polishing, the Ra values had no statistical difference when compared to the initial Ra (*p* > 0.05). For the DENSLD group, there were statistically significant differences (*p* < 0.05) in Ra at all times: initial (3.510 μm), after polishing (0.148 μm), and after brushing (0.600 μm). For the DENSLS group, there were statistically significant differences (*p* < 0.05) in the Ra values at all times: initial Ra (3.553 μm), Ra after polishing (0.232 μm), and Ra after brushing (0.406 μm). FXTSLD and FXTSLS groups did not show statistically significant differences at all times (*p* > 0.05).

Concerning wear values, DEN acrylic resin presented the highest wear values, for both SLD and SLS polishing systems, 31.21 (3.66) and 29.8 (4.3), respectively. The lowest values were obtained for FXT, also for SLD and SLS, 12.61 (1.24) and 12.7 (1.64). Meanwhile, among the bis-acryl resins, PT4 showed lower wear values, 13.95 (1.24) for SLD and 14.91 (2.26) for SLS when compared to ST3, 15.77 (1.32) for SLD and 15.4 (2.05).

**Figure 1:**
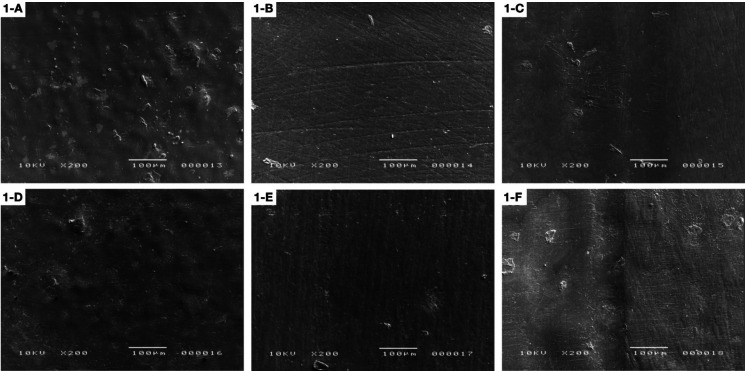
Protemp 4: A) Initial surface roughness (SLD); B) After polishing (SLD); C) After brushing (SLD); D) Initial surface roughness (SLS); E) After polishing (SLS); F) After brushing (SLS).

**Figure 2:**
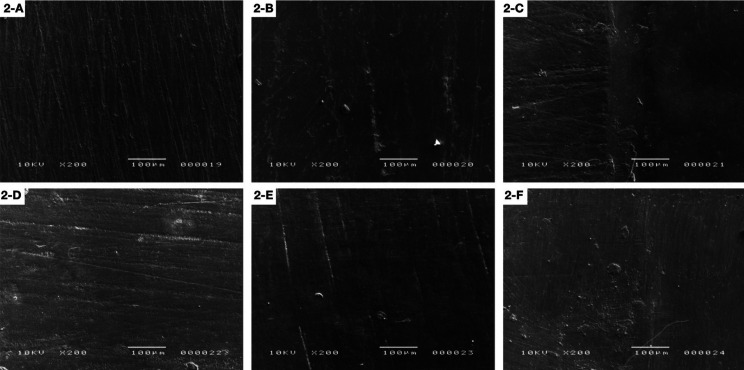
Structur 3: A) Initial surface roughness (SLD); B) After polishing (SLD); C) After brushing (SLD); D) Initial surface roughness (SLS); E) After polishing (SLS); F) After brushing (SLS).

The following SEM images show the differences among samples at three times: initial surface roughness (A), after polishing (B) and after brushing procedures (C) for SLD and initial surface roughness (D), after polishing (E) and after brushing procedures (F) for SLS polishing system. The PT4SLD group showed better polishing results when compared to the PT4SLS group (Fig. 1). ST3 groups presented grooves on initial surface roughness that became less evident after both types of polishing procedures, followed by a smoother surface after brushing (Fig. 2).

**Figure 3:**
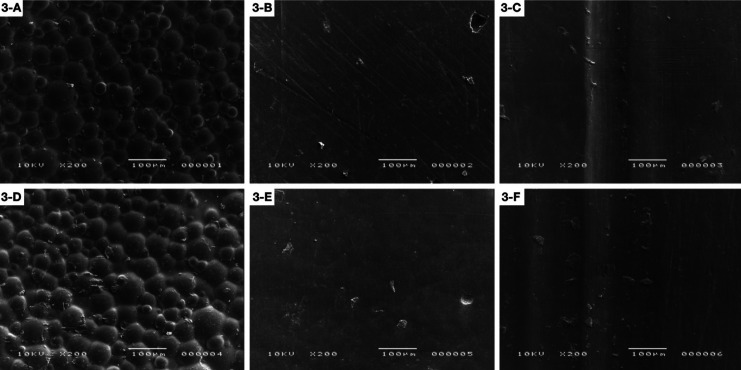
Dencor: A) Initial surface roughness (SLD); B) After polishing (SLD); C) After brushing (SLD); D) Initial surface roughness (SLS); E) After polishing (SLS); F) After brushing (SLS).

**Figure 4:**
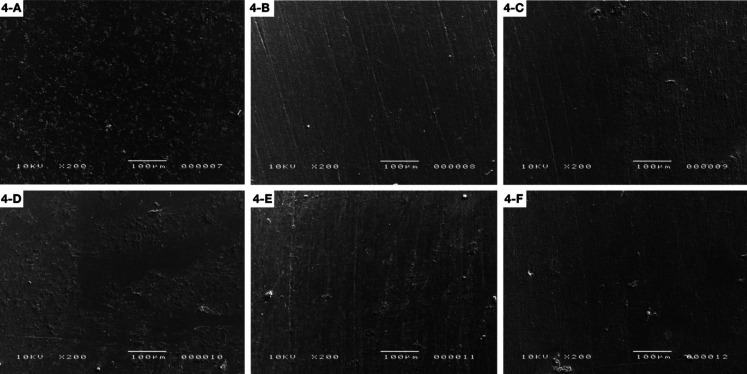
Z350XT: A) Initial surface roughness (SLD); B) After polishing (SLD); C) After brushing (SLD); D) Initial surface roughness (SLS); E) After polishing (SLS); F) After brushing (SLS).

For DENSLD group, it is possible to observe that there were grooves on the surface that were removed after brushing. While for DENSLS, the grooves showed up after brushing (Fig. 3). The FXTSLD and FXTSLS groups showed grooves on the entire surface after polishing and the roughness is more evident after brushing for this material (Fig. 4).

## Discussion

Surface imperfections and increased roughness of interim resin-based restorations may compromise clinical longevity, favoring staining, plaque accumulation and gingival inflammation (23,24). Therefore, adequate finishing and polishing procedures are necessary not only for aesthetics purposes, but to ensure smoothness, brightness and preservation of periodontal and pulpal health. Few studies have been carried out on polishing methods comparing provisional materials with different monomer compositions, such as PMMA, dimethacrylate-based and bis-acryl resins. In this study, surface roughness and wear of resin-based materials were evaluated after polishing with two different polishing systems (discs and spirals) and after simulated brushing. In addition, morphologic evaluation was performed through SEM images. After analyzing the results of the study, the null hypothesis was rejected, due to the significant differences observed among the various resin-based materials and the polishing systems.

The samples in this study were polished 24 hours after preparation in order to provide better marginal seal and to avoid plastic deformation (8,25), except for the bis-acryl samples, in which the polishing procedure was performed immediately after the setting time, following the manufacturer’s instructions. The polishing procedures in this study were performed with SLS, a two-step system, in which the first spiral (pre-polishing) is composed by aluminum oxide and the second is made with diamond particles, and SLD are flexible discs with aluminum oxide coating.

Both polishing systems evaluated were able to reduce surface roughness, however, SLS polishing system presented higher roughness for all materials tested compared to SLD. Some authors propose that the abrasive particles of the polishing material must be harder than the filler particle on the resin-based material, if not, the polishing will only expose the filler particles on the surface (26,27), which explains the good performance of SLD and SLS systems, since aluminum oxide hardness (Mohs) is considerably higher than many filler particles used in resin materials (28).

The clinically accepted surface roughness threshold for composite resin with respect to plaque accumulation restorations is 0.2 µm (29), In the present study, Ra values after polishing ranged between 0.039 and 0.284 µm for FXT after polishing with SLD and bis-acryl resin PT4 polished with the spiral system, respectively. After polishing procedures, both bis-acryl resins evaluated and DEN acrylic resin did not meet the criterion, but only when the system used was SLS. These results are in consonance with previous studies that confirmed that aluminum oxide discs like SLD are able to produce better surface smoothness (13,26,30,31).

As observed, monomer composition influenced the results of the presented study. PMMA-based resin (DEN) exhibited high surface roughness and the highest wear values, for both polishing systems. Some previous studies have reported lower wear resistance and surface microhardness for those self-curing conventional methacrylate resins and it may be explained due to the low molecular weight and linear monomers and, consequently, reduced rigidity (10). Contrarily, FXT resin composite demonstrated the lowest values for both surface roughness after polishing and wear after simulated brushing. The properties of restorative materials are highly influenced by type, size and amount of filler particles and nanofill and nanohybrid composites are considered highest development in terms of filler content (32), and the type of filler is also associated to mechanical properties like polishability and resistance to wear (33). FXT contains 78.5wt% of combination of 20 nm silica nanoparticles and 0.6-1.4 µm zirconia-silica nanoagglomerates (Table 1), and homogenously distributed smaller filler particles indicate smoother surfaces after polishing (34). These findings agree with previous studies, that demonstrated that surface roughness is correlated to the size of filler particles (35,36).

Besides some bis-acryl manufacturer’s instructions, in which only removing of the surface layer with a gauze soaked in alcohol is recommended, usually cervical, occlusal and proximal adjustments with burs or sandpapers may be required (7,12). The present study demonstrated that, among the bis-acryl resins tested, only ST3 presented Ra values below 0.2 µm, supporting manufacturer’s recommendations that polishing is unnecessary for this material. However, PT4 bis-acryl resin showed significantly lower roughness values after polishing.

Artificial aging tests are important to evaluate the longevity of resin-based materials (37). In furtherance of simulating abrasion on the surface of the samples and evaluate wear, 50.000 cycles were performed on a brushing machine, which corresponds to approximately five years in oral environment (38,39). The amount of filler particles, the organic matrix and the coupling agent are considered crucial factors regarding to resin-based materials wear (40). In this study, after simulated brushing, FXT presented the lowest wear values, but comparable to PT4. The resistance to wear of FXT and PT4 may be related to their filler particles, since both contain silane-treated silica, that can improve monomer conversion and mechanical properties (41), and it has positive effects on wear resistance (42).

## Conclusions

Within the limitations of this current study, it was concluded that:

1. Sof-Lex Pop-On discs system promoted better surface polishing in comparison to Sof-Lex spirals for all tested materials.

2. Z350XT resin composite groups presented the lowest roughness values for all evaluated periods, followed by bis-acryl resins groups, meanwhile Dencor acrylic resin groups exhibited the highest.

3. After simulated brushing, all groups showed increased roughness. Z350XT presented the lowest wear values, followed by bis-acryl resins Protemp 4 and Structur 3, and Dencor acrylic resin showed the highest wear.
